# Supplementary material for: Exploring Natural Alkaloids from Brazilian Biodiversity as Potential Inhibitors of the Aedes aegypti Juvenile Hormone Enzyme: A Computational Approach for Vector Mosquito Control
Source: Molecules. 2023 Sep 29;28(19):6871. doi: 10.3390/molecules28196871 (PMC10574778; doi:10.3390/molecules28196871)
Supplement: Supplementary file 1 [file molecules-28-06871-s001.zip › molecules-2527705-supplementary.pdf]

Exploring Natural Alkaloids from Brazilian Biodiversity as Potential Inhibitors of the *Aedes aegypti* Juvenile Hormone Enzyme: A Computational Approach for Vector Mosquito Control

Renato Araújo da Costa [1,2,*], Andréia do Socorro Silva da Costa [1], João Augusto Pereira da Rocha [3], Marlon Ramires da Costa Lima [2], Elaine Cristina Medeiros da Rocha [3], Fabiana Cristina de Araújo Nascimento [1], Anderson José Baia Gomes [2], José de Arimatéia Rodrigues do Rego [1] and Davi do Socorro Barros Brasil [1]

[1] Laboratory of Biosolutions and Bioplastics of the Amazon, Graduate Program in Science and Environment, Institute of Exact and Natural Sciences, Federal University of Pará (UFPA), Belém 66075-110, PA, Brazil; ac165051@gmail.com (A.d.S.S.d.C.); fabiananascimento987@gmail.com (F.C.d.A.N.); jr2rego@gmail.com (J.d.A.R.d.R.); davibb@ufpa.br (D.d.S.B.B.)

[2] Laboratory of Molecular Biology, Evolution and Microbiology, Federal Institute of Education,
Science and Technology of Pará (IFPA) Campus Abaetetuba, Abaetetuba 68440-00, PA, Brazil;
ramireslima9774@gmail.com (M.R.d.C.L.); anderson.gomes@ifpa.edu.br (A.J.B.G.)

[3] Graduate Program in Chemistry, Federal University of Pará (UFPA), Belém 66075-110, PA, Brazil;
joao.rocha@ifpa.edu.br (J.A.P.d.R.); elaine.rocha@ifpa.edu.br (E.C.M.d.R.)

[4] Laboratory of Modeling and Computational Chemistry, Federal Institute of Education,
Science and Technology of Pará (IFPA) Campus Bragança, Bragança 68600-000, PA, Brazil

* Correspondence: renato.costa@ifpa.edu.br; Tel.: +55-91-985-484-622

Table S1: codes and interaction energies for all NuBBEDB alkaloids used in this study.

Compouds	PLPchem
NuBBE_16	91.5718
NuBBE_17	96.2742
NuBBE_18	61.3529
NuBBE_19	79.1863
NuBBE_20	79.5186
NuBBE_21	84.0892
NuBBE_22	84.7095
NuBBE_23	81.4468
NuBBE_24	77.0972
NuBBE_25	74.9031
NuBBE_27	77.6277
NuBBE_28	89.2420
NuBBE_40	68.4018

**NuBBE_41**	57.7215
**NuBBE_42**	70.1506
**NuBBE_43**	64.1775
**NuBBE_44**	48.9001
**NuBBE_45**	89.6063
**NuBBE_79**	83.1718
**NuBBE_80**	83.5053
**NuBBE_81**	85.2822
**NuBBE_97**	50.6861
**NuBBE_98**	62.5564
**NuBBE_105**	60.1450
**NuBBE_163**	44.0750
**NuBBE_164**	56.6799
**NuBBE_165**	41.8523
**NuBBE_215**	49.3100
**NuBBE_216**	62.5877
**NuBBE_299**	99.0613
**NuBBE_300**	97.1695
**NuBBE_301**	102.0825
**NuBBE_302**	93.9900
**NuBBE_303**	103.6489
**NuBBE_305**	92.4838
**NuBBE_306**	99.8558
**NuBBE_308**	86.5153
**NuBBE_326**	31.6051
**NuBBE_327**	23.7938
**NuBBE_328**	28.1316
**NuBBE_329**	42.6629
**NuBBE_373**	75.1925
**NuBBE_375**	85.1626
**NuBBE_408**	93.1023
**NuBBE_409**	70.8143
**NuBBE_427**	94.8389
**NuBBE_452**	88.3065
**NuBBE_453**	94.9499
**NuBBE_454**	100.1084
**NuBBE_456**	88.7836
**NuBBE_457**	87.3948
**NuBBE_532**	40.0143
**NuBBE_533**	24.9976
**NuBBE_547**	79.3221
**NuBBE_548**	69.2271
**NuBBE_549**	74.2708
**NuBBE_550**	74.4697
**NuBBE_596**	59.0956
**NuBBE_597**	65.0915
**NuBBE_598**	60.9971
**NuBBE_599**	52.6367
**NuBBE_600**	60.3971
**NuBBE_601**	55.2074

**NuBBE_634**	75.4550
**NuBBE_934**	58.5532
**NuBBE_935**	62.5920
**NuBBE_936**	65.9169
**NuBBE_937**	62.2096
**NuBBE_939**	65.2645
**NuBBE_941**	50.3615
**NuBBE_942**	46.4380
**NuBBE_943**	53.7081
**NuBBE_944**	60.3344
**NuBBE_955**	59.1110
**NuBBE_964**	61.4795
**NuBBE_965**	61.1504
**NuBBE_971**	57.6833
**NuBBE_1065**	62.2584
**NuBBE_1066**	58.3002
**NuBBE_1067**	64.9205
**NuBBE_1070**	50.8003
**NuBBE_1074**	72.3634
**NuBBE_1075**	49.0780
**NuBBE_1076**	44.9374
**NuBBE_1077**	34.9633
**NuBBE_1078**	29.9825
**NuBBE_1079**	27.2473
**NuBBE_1080**	40.6868
**NuBBE_1081**	32.2828
**NuBBE_1086**	81.8060
**NuBBE_1087**	76.6251
**NuBBE_1090**	36.2923
**NuBBE_1091**	61.7283
**NuBBE_1092**	44.0802
**NuBBE_1093**	40.8333
**NuBBE_1094**	52.4233
**NuBBE_1100**	60.9544
**NuBBE_1101**	47.6352
**NuBBE_1102**	52.5357
**NuBBE_1105**	111.3446
**NuBBE_1106**	107.2860
**NuBBE_1107**	113.0490
**NuBBE_1108**	101.3099
**NuBBE_1109**	58.2763
**NuBBE_1118**	78.6546
**NuBBE_1135**	73.9671
**NuBBE_1140**	18.1836
**NuBBE_1141**	36.3635
**NuBBE_1159**	70.8893
**NuBBE_1164**	74.2091
**NuBBE_1165**	78.3560
**NuBBE_1171**	54.9169
**NuBBE_1235**	51.2458

**NuBBE_1236**	48.6133
**NuBBE_1237**	35.6859
**NuBBE_1238**	44.2450
**NuBBE_1239**	69.6925
**NuBBE_1240**	74.4020
**NuBBE_1247**	74.7271
**NuBBE_1248**	72.4692
**NuBBE_1258**	61.0017
**NuBBE_1286**	55.9508
**NuBBE_1299**	69.1919
**NuBBE_1336**	46.4143
**NuBBE_1344**	46.1492
**NuBBE_1345**	57.2705
**NuBBE_1358**	43.7837
**NuBBE_1359**	25.2590
**NuBBE_1360**	31.2519
**NuBBE_1361**	38.7608
**NuBBE_1362**	51.0718
**NuBBE_1363**	69.5151
**NuBBE_1400**	78.7434
**NuBBE_1432**	22.6011
**NuBBE_1433**	54.8033
**NuBBE_1434**	49.7775
**NuBBE_1435**	63.8275
**NuBBE_1436**	58.7437
**NuBBE_1437**	50.2410
**NuBBE_1439**	66.0234
**NuBBE_1471**	61.7559
**NuBBE_1474**	35.5881
**NuBBE_1475**	49.4870
**NuBBE_1476**	32.6386
**NuBBE_1477**	46.9819
**NuBBE_1481**	29.9857
**NuBBE_1483**	26.5708
**NuBBE_1484**	44.8086
**NuBBE_1502**	66.6401
**NuBBE_1570**	60.6754
**NuBBE_1571**	46.0033
**NuBBE_1572**	45.0811
**NuBBE_1579**	48.2623
**NuBBE_1596**	61.6043
**NuBBE_1597**	73.1463
**NuBBE_1598**	70.6524
**NuBBE_1599**	87.5175
**NuBBE_1613**	40.4336
**NuBBE_1614**	23.6020
**NuBBE_1618**	66.1295
**NuBBE_1620**	54.6509
**NuBBE_1626**	52.4960
**NuBBE_1634**	66.7338

**NuBBE_1669**	50.6136
**NuBBE_1674**	44.9252
**NuBBE_1675**	67.5693
**NuBBE_1711**	52.8108
**NuBBE_1757**	43.9795
**NuBBE_1758**	25.3684
**NuBBE_1783**	63.3243
**NuBBE_1785**	13.6782
**NuBBE_1786**	38.1255
**NuBBE_1853**	59.9676
**NuBBE_1854**	57.9484
**NuBBE_1951**	37.5217
**NuBBE_1953**	64.6025
**NuBBE_1955**	71.7786
**NuBBE_1956**	68.9096
**NuBBE_1957**	66.8223
**NuBBE_1960**	30.2192
**NuBBE_1961**	54.3417
**NuBBE_1962**	2.8563
**NuBBE_1964**	62.9801
**NuBBE_2014**	50.9443
**NuBBE_2061**	29.1992
**NuBBE_2062**	44.0082
**NuBBE_2063**	66.9972
**NuBBE_2064**	52.4294
**NuBBE_2065**	50.0326
**NuBBE_2066**	29.8477
**NuBBE_2067**	31.9148
**NuBBE_2084**	74.0207
**NuBBE_2090**	87.1343
**NuBBE_2091**	90.6712
**NuBBE_2092**	90.7519
**NuBBE_2093**	80.8707
**NuBBE_2094**	84.5341
**NuBBE_2202**	70.5923
**NuBBE_2203**	56.4328
**NuBBE_2227**	36.3958
**NuBBE_2228**	43.4907
**NuBBE_2301**	20.6216
**NuBBE_2302**	12.2172
**NuBBE_2303**	6.3109
**NuBBE_2304**	29.9204
**NuBBE_2365**	60.0978
**NuBBE_2366**	-14.8581
**NuBBE_2367**	47.4816
**NuBBE_2368**	48.1225
**NuBBE_2406**	46.5884
**NuBBE_2407**	38.9733
**NuBBE_2408**	52.2652
**NuBBE_2411**	42.9995

**NuBBE_2419**	47.0298
**NuBBE_2420**	39.7510
**NuBBE_2421**	41.3075
**NuBBE_2422**	22.2871
**NuBBE_2424**	83.7963
**NuBBE_2425**	79.3734
**NuBBE_2436**	58.4336
**NuBBE_2484**	41.7588
